# An unusual pattern of endometrial involvement: superficial spreading squamous cell carcinoma of the cervix

**DOI:** 10.3389/fonc.2024.1456297

**Published:** 2024-10-01

**Authors:** Xiaolin Jiang, Zhirong Han, Zhiping Chun, Bingyang Wen, Tingan Chen

**Affiliations:** ^1^ Department of Pathology, Guangyuan Central Hospital, Guangyuan, China; ^2^ Department of Pathology, Guangyuan City Hospital of Traditional Chinese Medicine, Guangyuan, China

**Keywords:** superficial spreading, squamous cell carcinoma, HSIL, cervix, endometrium

## Abstract

**Background:**

Cervical squamous cell carcinoma (SCC) is the most common type of cervical carcinoma. Usually, the cancer metastasizes through lymphatic or hematogenous dissemination. However, it is uncommon for a superficial spreading of cervical cancer to reach the endometrium, fallopian tubes, and the ovaries.

**Objectives:**

In the present study, we report 15 cases of superficial spreading SCC and discuss the possible mechanism involved.

**Methods:**

We collected 15 samples diagnosed by histopathology after surgery. Immunostaining, which included P16, P63, CD138, CD34, D2-40, and Ki-67, were performed for all samples.

**Results:**

All patients were postmenopausal or perimenopausal women. The commonest clinical presentation was vaginal bleeding in 66.67%. All patients were infected with HPV 16. The endometrium was replaced by high-grade squamous intraepithelial lesion (HSIL), which involved the endometrial gland, even squeezing into the myometrium and forming SCC. Bilateral fallopian tubes and ovaries involvement was in 1/15. A total of 10/15 (66.67%) of the women had disease of stage 1B or less. All SCCs were moderately or poorly differentiated. Immunohistochemistry revealed that the tumor cells were positive for P63 and P16, with a high Ki-67 labeling index. There was CD138 positive expression in varying degrees, which was strongly and diffusely expressed in 6/15 (40.00%).

**Conclusion:**

Superficial spread of cervical cancer towards the endometrium is a rare but cognizable phenomenon, and a guideline for the management of these cases has not been established. Our present findings suggest that multiple factors may interact with each other simultaneously, contributing to this rare disease.

## Introduction

Cervical cancer is the fourth most common cancer among women globally (1). Normally, it extends downward into the vagina or, laterally, invading the parametrial tissue, and it also metastasizes through the lymphatic glands or by blood dissemination. Few cases with superficial spread to the uterine endometrium, fallopian tubes, and ovaries have been observed. Cervical SCC, spreading superficially to the inner surface of the uterus and replacing the endometrium with carcinoma cells, is called superficial spreading SCC (2). It is not included in the 2020 (fifth edition) World Health Organization (WHO) Classification of Female Genital Tract Tumors or the 2018 FIGO (International Federation of Gynecology and Obstetrics) cervical cancer staging system. It is undetermined if this kind of superficial spread changes the stage, management, and prognosis.

Notably, superficial spreading SCC should be distinguished from cervical squamous cell carcinoma directly invading the uterine wall and primary endometrial SCC (PESCC). The diagnostic criteria for PESCC were established by Fluhmann (3) in 1953 and included the following: (a) no evidence of coexisting endometrial adenocarcinoma or primary cervical SCC, (b) no connection between the endometrial tumor and squamous epithelium of the cervix, or (c) no connection between any existing cervical *in situ* carcinoma and independent endometrial neoplasm.

In the literature, only a few dozen cases have been reported. In the present study, we reported 15 cases of superficial spreading SCC of the cervix involving the endometrium and discussed the possible mechanism of this unusual spreading form of cervical SCC.

## Materials and methods

Ethical approval was obtained from the Guangyuan Central Hospital Ethics Review Committee. Written informed consent for participation was not required for this study in accordance with the national legislation and the institutional requirements. In this retrospective study, we collected 15 samples in the Department of Pathology of Guangyuan Central Hospital and Guangyuan City Hospital of Traditional Chinese Medicine from January 2020 to December 2023. All patients underwent surgery.

The pathology specimens in all cases were fixed in 10% neutral formalin. The samples were embedded in paraffin blocks, cut at 4-μm thickness, and stained with standard hematoxylin and eosin (H&E). Immunostaining was performed according to the manufacturer’s instructions, and the tested antibodies included P16, P63, CD138, CD34, D2-40, and Ki-67 (Zhongshan, Beijing, China).

All histopathological slides from each patient were reviewed, and superficial spreading SCC was confirmed by two professional and experienced pathologists with consistent diagnosis independently and in duplicate. Focal LVSI is defined by the presence of a single focus around the tumor, substantial LVSI as multifocal or diffuse arrangement of LVSI, or the presence of tumor cells in four or more lymphovascular spaces (4). The prognosis of the patients was defined as survival period in months after surgery and histological diagnosis.

### Statistical analysis

Statistical analysis and data management were conducted using SPSS, version 20 (SPSS, Chicago, IL, USA). Categorical data were expressed as absolute or relative frequencies, and continuous data were expressed as mean ± SD.

## Results

The study involved 15 patients. The age of all patients was over 50 years (age, 62.86 ± 9.52 years; range, 50–81 years). Except for patient F1 and F11, who were perimenopausal women, all other patients were postmenopausal women (menopausal age, 49.77± 3.85 years; menopausal duration, 14.23 ± 7.96 years). Most of the women were 50–60 years (53.33%). There was a wide spectrum of clinical presentations, with the most common clinical presentation being vaginal bleeding (10/15, 66.67%), followed by cervical stenosis (5/15, 33.33%), pyometra (4/15, 26.67%), genital discharge (3/15, 20.00%), hydrometra (2/15, 13.33%), paramenia (1/15, 6.67%), and abdominal pain (1/15, 6.67%). One woman may have more than one complaint. All patients were infected with HPV 16. Increased squamous cell carcinoma antigen (SCC-Ag) patients were 6/7 (85.71%). Comorbidities and personal history are in shown **Table 1**. Seven of the 15 (46.67%) patients had history of miscarriage, including spontaneous and induced abortion. All patients had two or more children. There were five leiomyoma of the uterus and two adenomyosis.

**Table 1:** Clinical and biological data from patients with superficial spreading SCC.

Patient no.	Age (years)	Clinical presentation	HPV	Menopausal age/duration	Comorbidities	Personal history and childbearing history	SCC-Ag
F1	51	Vaginal bleeding	16	Perimenopause	Ectopic pregnancy	More than 20 years of smoking; G:8, P:1	ND
F2	75	Vaginal bleeding	16	50/25	LC	G:3, P:3	ND
F3	71	Pyometra; Vaginal bleeding; Cervical stenosis	16	50/21	NO	More than 20 years of rheumatoid disease; G:2, P:2	ND
F4	65	Abdominal pain; Vaginal bleeding	16	49/16	Hypertension	IUD; G:3, P:1	ND
F5	55	Vaginal bleeding	16	50/5	NO	G:4, P:3	ND
F6	53	Vaginal bleeding	16	49/4	Hypertension	IUD; G:5, P:2	Increased
F7	59	Vaginal bleeding; Hydrometra	16	52/7	NO	40 years of drinking;G:4, P:1	Increased
F8	58	Vaginal bleeding; Hydrometra Cervical stenosis	cp8304/16/39	49/9	NO	G:2, P:2	ND
F9	59	Vaginal bleeding; Cervical stenosis	16	43/16	LC;	40 years of smoking; Bilateral tubal ligation; G:2, P:2	Normal
F10	66	Genital discharge; Pyometra	16	59/7	Hypertension Tuberculosis	G:6, P:3	ND
F11	50	Paramenia	16	Perimenopause	Myocardial ischemia	G:3, P:3	ND
F12	65	Genital discharge;	16	52/13	NO	G:2, P:2	Increased
F13	81	Vaginal bleeding Cervical stenosis	16	52/29	Diabetes; Fungal pneumonia	Bilateral tubal ligation; G:9, P:9	Increased
F14	76	Genital discharge; Pyometra Cervical stenosis	16/31/58	45/21	Hypertension	G:5, P:5	Increased
F15	59	Pyometra	16	47/12	NO	G:3, P:2	Increased

IUD, intrauterine ring; LC, laparoscopic cholecystectomy; SCC-Ag, squamous cell carcinoma antigen; ND, not done.

**Figure 1:**
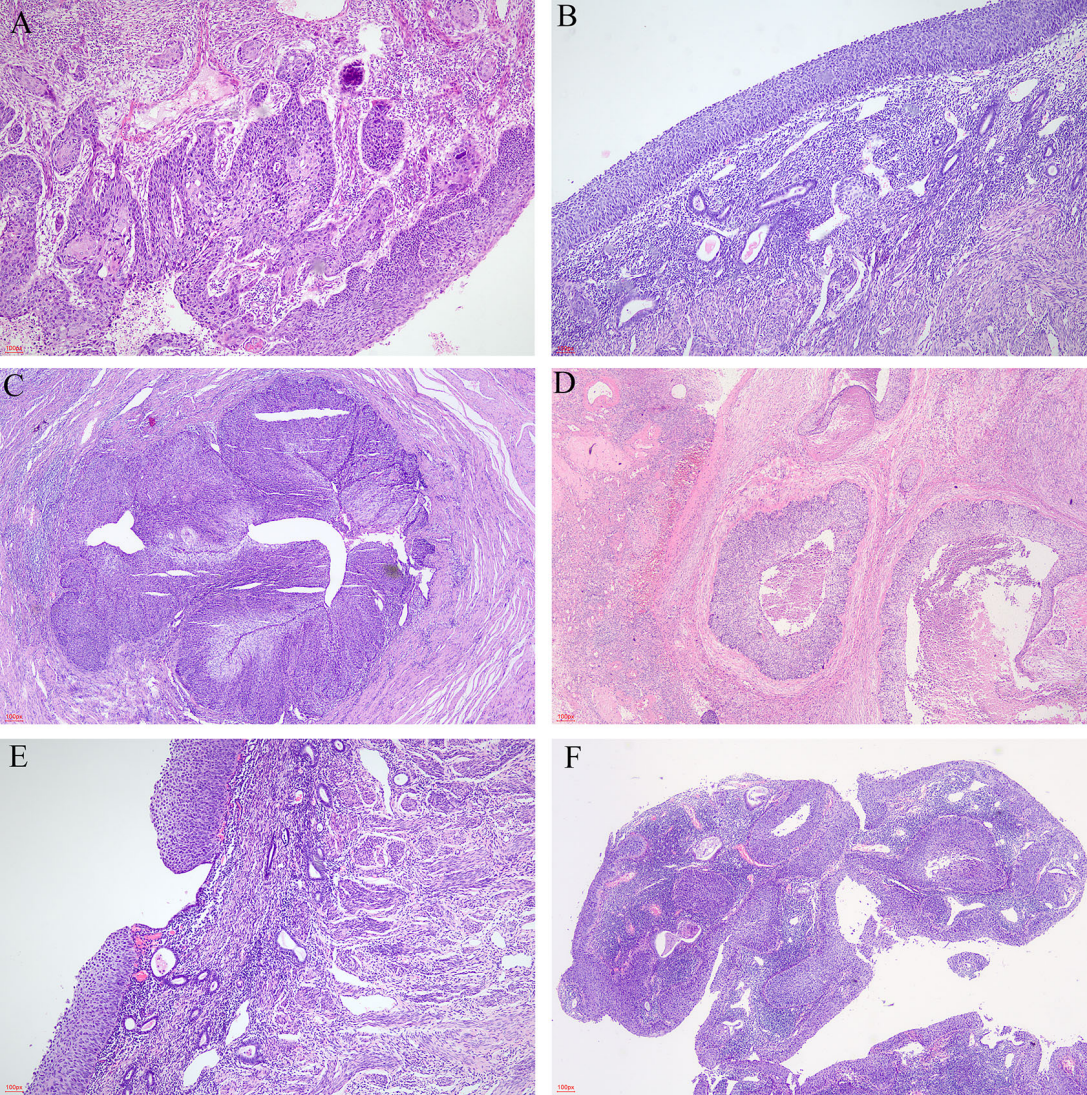
The microscopic appearance of lesions. **(A)** Cervical lesion (HE, ×40). **(B)** Superficial spreading atypical squamous cells in the endometrium (HE, ×40). **(C)** Superficial spreading SCC in bilateral fallopian tubes (HE, ×20). **(D)** Superficial spreading SCC in bilateral ovaries (HE, ×20). **(E)** Intermittent or skipping superficial spreading in endometrium (HE, ×40). **(F)** Endometrial polyps covered by HSIL (HE, ×20).

Under microscopic examination, the cervix showed high-grade squamous intraepithelial lesion (HSIL) and/or SCC with chronic inflammation. These atypical squamous cells exhibited high nuclear to cytoplasmic ratio, rounded nuclei with distinct nucleoli, and high mitotic activity (**Figure 1A**). The commonest histological subtype of cervical lesion was SCC with HSIL in 12/15 (80.00%) cases, SCC without HSIL in 2/15 (13.33%), and one case of HSIL with microinvasive SCC (6.67%).

The endometrium was also replaced by HSIL (**Figure 1B**), which involved the endometrial gland, even squeezing into the myometrium and forming SCC. This endometrial lesion was directly contiguous to the cervical lesion. The extensional lesion of the 15 superficial spreading SCC cases are as follows: endometrial HSIL was in 6/15 (40.00%), endometrial HSIL with microinvasive in 3/15 (20.00%), and endometrial HSIL with SCC in 6/15 (40.00%). Vaginal HSIL was 2/15 (F7 and F10), bilateral fallopian tubes and ovaries involvement was in 1/15 (F12) (**Figures 1C, D**), and pelvic lymph nodes metastasis was in 1/15 (F1). An interesting finding was that F7 had SCC and HSIL, which had intermittent or skipping superficial spreading in the endometrium (**Figure 1E**). The patient F8 underwent laparoscopic hysterectomy and bilateral adnexectomy. By macroscopic examination, we found that the endometrium was slightly thicker with an adherent polyp-shaped lump extending to the right uterine cornua. The pathology results of F8 showed SCC and HSIL within the cervix, microinvasive and HSIL in the endometrium, and endometrial polyps covered by HSIL (**Figure 1F**). Lymph-vascular space invasion (LVSI) was also discovered in 11/15. Focal LVSIs were 5/15. Substantial LVSIs were 6/15.

The patients were classified by FIGO 2018 staging system as follows: 6.67% (1/15) patients with stage IA1, 40.00% (6/15) patients with stage IB1, 6.67% (1/15) with stage IB2, 13.33% (2/15) with stage IB3, 20.00% (3/15) with stage IIA1, 6.67% (1/15) with stage IIB, and 6.67% (1/15) with stage IIIC1. A total 10/15 (66.67%) of the women had disease of stage 1B or less. Thus, despite the lower stage in the cervix, the tumor extended cephalad, indicating an inherent propensity for cephalad extension rather than infiltration as is the usual case.

Of the SCC tumors, 3/14 (21.43%) had poorly differentiated tumors, and 11/14 (78.57%) had moderately differentiated tumors. Majority of the patients were treated with radiotherapy or chemotherapy after surgery, including five with chemoradiotherapy and six with chemotherapy.

**Figure 2:**
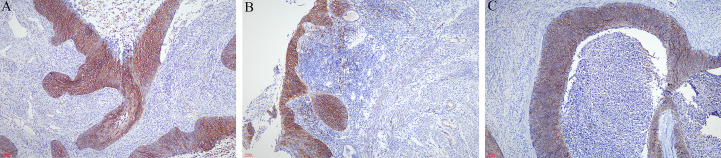
Immunohistochemical staining of CD138. **(A)** CD138 was strongly and diffusely expressed in the cervix (×40). **(B)** CD138 was strongly expressed in the neoplastic squamous cells spreading in the endometrium (×40). **(C)** Superficial spreading SCC in the ovary also expressed CD138 (×40).

The results of immunohistochemical staining revealed that carcinoma cells, whether in the cervix, endometrium, ovaries, or fallopian tubes, were diffusely positive for P63 and p16. The Ki-67 labeling index was 40%–90%. CD34 and D2–40 staining verified carcinoma invasion in the cervical stromal lymph vascular space. There was positive expression in varying degrees of CD138 in the cervix (**Figure 2A**) and extended lesion (**Figures 2B, C**). CD138 was strongly and diffusely expressed in 6/15 (40.00%).

Survival data were available for all cases (**Table 2**). The median duration of follow-up was 13 months (range, 6–49 months). All patients were alive after surgery with no evidence of clinical recurrence or metastasis.

**Table 2:** Superficial spreading SCC of uterine cervix involving the endometrium and other extended lesion.

Patient No.	Cervical lesion	Extension of lesion	LVSI	Differentiation degree	FIGO Stage	Treatment	Follow-up (months)
F1	SCC	Endometrium (HSIL) Pelvic lymph nodes metastasis	Substantial	Moderately	III C1	Chemoradiotherapy	NED (15)
F2	SCC, HSIL	Endometrium (HSIL)	Negative	Moderately	II A1	Chemotherapy	NED (12)
F3	SCC, HSIL	Endometrium (HSIL, Microinvasive)	Substantial	Moderately	I B1	NO	NED (11)
F4	SCC	Endometrium (HSIL)	Focal	Moderately	I B1	Chemotherapy	NED (8)
F5	SCC, HSIL	Endometrium (HSIL; Microinvasive)	Negative	Moderately	II B	Chemotherapy	NED (36)
F6	SCC, HSIL	Endometrium (HSIL)	Focal	Moderately	I B2	Chemoradiotherapy	NED (10)
F7	SCC, HSIL	Endometrium (HSIL, SCC) Vagina (HSIL)	Substantial	Poorly	I B3	Chemotherapy	NED (9)
F8	SCC, HSIL	Endometrium (HSIL, Microinvasive)	Substantial	Moderately	II A1	Chemoradiotherapy	NED (10)
F9	SCC, HSIL	Endometrium (HSIL)	Negative	Moderately	I B1	Chemotherapy	NED (13)
F10	HSIL, Microinvasive	Endometrium (HSIL, SCC) Vagina (HSIL)	Focal	–	IA1	NO	NED (21)
F11	SCC, HSIL	Endometrium (HSIL, SCC)	Substantial	Moderately	I B3	Chemoradiotherapy	NED (22)
F12	SCC, HSIL	Endometrium (HSIL, SCC) Bilateral fallopian tubes and ovaries (HSIL, SCC)	Substantial	Poorly	I B1	Chemoradiotherapy	NED (18)
F13	SCC, HSIL	Endometrium (HSIL, SCC)	Focal	Poorly	I B1	NO	NED (32)
F14	SCC, HSIL	Endometrium (HSIL, SCC)	Negative	Moderately	I B1	NO	NED (49)
F15	SCC, HSIL	Endometrium (HSIL)	Focal	Moderately	II A1	Chemotherapy	NED (6)

NED, no evidence of disease.

## Discussion

In 1900, Cullen first discovered a case of cervical SCC that spread to the entire endometrium; since then, only few cases have been reported, with an occurrence of 0.7% (5). Among these rare cases, the endometrium is the most common site for metastasis. Various types of cervical cancer can superficially spread to the endometrium, fallopian tube, and ovaries. Among these types of cervical cancer, squamous cell carcinoma (91.11%) has the highest frequencies (6).

Since the 1960s, scholars have discussed the potential risk factors that may contribute to upward metastasis, such as long-term estrogen usage, vitamin A deficiency, HPV infection, senile endometrium, pyometra, and radiotherapy (5); among them, some have persisted until today, namely, advanced age, cervical stenosis, and pyometra (7). The most common clinical presentation is vaginal bleeding and pyometra in the previous literature (8).

In the previous report, the age of all patients was over 50 (2), and all were menopausal or post-menopausal (7). The most common clinical presentation was genital bleeding (2), which is similar to our result. Cervical stenosis was proposed to be a reason for endometrial rather than the lateral extension (9). However, our analysis indicates that among 15 cases with information about the cervical lesion, only five (33.33%) women had stenosis; hence, this may not be the only reason for the superficial spread of the tumor. In postmenopausal women, cervical stenosis is easily formed after cervical treatment due to the loss of periodic abscission, and it encloses the uterine cavity and accelerates the pyometra. Anne Chao et al. (10) also reported a case with fatal pyometra in a 60-year-old patient whose pathology also revealed cervical HSIL that progressed into SCC in the endometrium. In this study, 4/15 (26.67%) patients had pyometra.

CD 138 is a key cell surface adhesion molecule. It is a cell surface heparin sulfate proteoglycan that is responsible for cell–cell and cell–extracellular matrix interaction. It has been proposed that this may be one possible reason for the superficial spread of this tumor rather than infiltration due to retained CD138 expression in these cells (2). Expression of CD138 may participate in superficial spread by cell–cell interactions. Two authors reported that the superficial spreading cells were strongly positive for CD138 in superficial carcinoma cells in both the cervix and endometrium (2, 11). In our study, CD138 was positively expressed in varying degrees, whereas it was strongly expressed in 40.00%. The report is not entirely consistent with our conclusion, which may be related to the rare sample and limited information about CD138.

As the most common malignant tumor in the female reproductive system, cervical SCC (>90–95%) is HPV associated in the large majority of cases. In one of the studies reviewed, all the samples analyzed were HPV 16 positive (10). Consistent with our case, all samples were positive for HPV 16. This suggests that persistent HR-HPV infection is a key factor in the development of superficial spreading cervical SCC.

Another proposed mechanism is the transformation of endometrial cells to squamous rather than actual spread. P16 over expression and HPV linkage have been proposed to be responsible for transforming the reserve cells in the endometrium misconceived as the superficial spread (12). Of the patients with endometrial cancer, 90% have abnormal vaginal bleeding as the first main symptom, most frequently during the postmenopausal period. Due to a new diagnostic classification centered on molecular and immunohistochemical indicators, the risk classification is much more accurate. On the basis of the outcomes of the Cancer Genome Atlas, and the ProMisE (Proactive Molecular Risk Classifier for EC), endometrial cancer is divided into four subgroups: POLEmut, p53 wild type (low copy number—CNL—or nonspecific molecular profile—NSMP), p53 null/missense mutations (high copy number), and mismatch repair deficient (MMRd) (13). But in another study, Kushima et al. (14) studied the loss of heterozygosity markers in the endometrial cells in five women indicating that a monoclonal origin of endometrial and cervical tumors supported spread from the cervix rather than endometrial cell transformation.

It has been reported that the bilateral tube and ovary were directly spread from HPV-associated superficial uterine cervical squamous carcinoma through the endometrium and the fallopian tube and lymph-vascular space invasion at the same (15). The mechanism may involve either LVSI moving to the ovarian hilum or ovary surface involvement of the endometrium and fallopian tubes, similar to double-hit ways hand in hand. LVSIs are known risk factors for recurrence in cervical cancer and are also predictive of lymph node metastasis. In our findings, all SCCs were moderately or poorly differentiated; we suggest that it may be related to superficial spread of cervical SCC.

It is currently difficult to draw any conclusion regarding optimal mechanism. Therefore, it is reasonable to infer that these factors often interact with each other simultaneously, contributing to this rare disease.

Deel et al. (16) postulate that in women with the localized cervical disease, the superficial spread to the endometrium and adnexa may not be associated with an inferior prognosis. Our patient was alive and well at 6 months after surgery. Perhaps due to the short follow-up time of our cases, the relationship between superficial spreading SCC and prognosis cannot be confirmed at present. To date, too few cases of superficial spreading SCC of the cervix have been reported to establish a conclusion regarding their treatment and prognosis. Perez et al. proposed that endometrial involvement of cervical SCC may be an indicator of poor prognosis and is usually diagnosed after hysterectomy; early detection is of extreme significance (17). It is wise to evaluate the endometrium thoroughly in every case of cervical cancer to offer the best management modalities to the patient. If untreated, this superficial spread may lead to the recurrence of the tumor. A careful evaluation and therapeutic strategy must be adopted for optimal disease-free outcomes.

Superficial spread of cervical cancer towards the endometrium is a rare but cognizable phenomenon, and a guideline for the management of these cases has not been established. In addition, the FIGO staging system has no descriptions for such a condition. Superficial spread of cervical cancer is a distinct entity. Our present findings suggest that multiple factors may interact with each other simultaneously, contributing to this rare disease. There are insufficient data to compare superficial spreading SCC of the cervix with other types of cervical cancer. More clinical cases are needed to identify additional prognostic factors and inform clinical practice guidelines on the management of this disease.

## Data Availability

The original contributions presented in the study are included in the article/supplementary material. Further inquiries can be directed to the corresponding author.
